# Prevalence of Hypothyroidism in Pregnant Women in India: A Meta-Analysis of Observational Studies

**DOI:** 10.1155/2021/5515831

**Published:** 2021-02-19

**Authors:** Vikas Yadav, Deepti Dabar, Akhil D. Goel, Mohan Bairwa, Akanksha Sood, Pankaj Prasad, Sanjay S. Agarwal, Sunil Nandeshwar

**Affiliations:** ^1^Atal Bihari Vajpayee Government Medical College, Vidisha, India; ^2^All India Institute of Medical Sciences, Bhopal, India; ^3^All India Institute of Medical Sciences, Jodhpur, India; ^4^All India Institute of Medical Sciences, New Delhi, India; ^5^St. Mary's Hospital, Oxford Road, Manchester, UK

## Abstract

**Introduction:**

This meta-analysis was conducted to estimate the prevalence of hypothyroidism among pregnant women in India.

**Methods:**

We searched PubMed, Web of Science, Scopus, Google Scholar, and Shodhganga (Indian thesis repository) for observational studies, providing prevalence of hypothyroidism among pregnant women in India. Systematic study selection and data extraction procedures were followed. Quality assessment of each study was done using JBI critical appraisal checklist. The random effects model was used for pooling the effect sizes. Publication bias was assessed using the funnel plot and rank correlation test. *I*^2^ statistics was used to measure heterogeneity across the studies. Heterogeneity in the pooled estimates was further explored with subgroup analyses and meta-regression analysis.

**Results:**

Sixty-one studies were found eligible and included in this review. The pooled estimate of the prevalence of hypothyroidism in pregnant women was 11.07% (95% CI: 8.79–13.84, *I*^2^ = 99%). Pooled prevalence estimates of subclinical and overt hypothyroidism are 9.51% (95% CI: 7.48–12.04, *I*^2^ = 98%) and 2.74% (95% CI: 2.08–3.58, *I*^2^ = 94%).

**Conclusion:**

We documented 11.07% pooled prevalence of hypothyroidism in pregnant women in India.

## 1. Introduction

Pregnancy has a significant effect on the thyroid gland and its functioning [1]. Hypothyroidism in pregnancy is defined as an increased TSH level in serum. Furthermore, based on free T4 levels, it is categorized into overt (lower free T4 levels) and subclinical hypothyroidism (normal free T4 levels) [2].

Worldwide, several studies have reported 1.5%–4% prevalence of hypothyroidism in pregnant women. Among them, 0.3% to 0.5% had overt hypothyroidism (OH), and the rest had subclinical hypothyroidism (SCH) [3–5]. In India, reports on the prevalence of maternal hypothyroidism ranged between 1.2% and 67.0% in various studies [6, 7].

Globally, the leading cause of hypothyroidism in pregnancy is iodine deficiency, and in iodine sufficient areas, most common cause is autoimmune thyroiditis [4, 5, 8]. Other common causes are radio-iodine therapy, thyroidectomy, congenital hypothyroidism, drug use (i.e., rifampicin and phenytoin) and any hypothalamic-pituitary disease [4, 5, 8]. Women with lower thyroid reserves preconceptually are often unable to cope with increased metabolic demands during pregnancy period and can enter into the hypothyroid state. Maternal thyroid hormone levels are critical to the fetus, especially in the first trimester due to inability to produce iodothyronines before ten weeks of gestation. This is the period when neurodevelopment of fetus can potentially be hampered due to deficiency of iodothyronines [9].

In pregnant women, untreated overt hypothyroidism is associated with gestational hypertension, abruptio placenta, anemia, gestational diabetes, and postpartum hemorrhage [10–13]. In overt hypothyroidism, there is also an increased risk of adverse birth outcomes. Frequently associated birth outcomes are spontaneous miscarriage, low birth weight, premature birth, fetal distress, perinatal death, and stillbirth [11, 14–19]. Overt hypothyroidism also has a detrimental impact on neurocognitive development of the fetus. Subclinical hypothyroidism might also have similar adverse effects, although the evidence is inconclusive [2]. Moreover, various studies have found that children born to mother with untreated hypothyroidism are at significantly higher risk of developing neuropsychological developmental disorders, which may manifest as lower IQ scores and other learning disabilities [20–22].

Earlier studies have also proved that most of these complications can be prevented by high-risk screening pregnant women for thyroid status and providing treatment in the form of levothyroxine (LT4) [2]. To effectively start such a screening program in India, there is a need to have a national-level estimate of the prevalence of this disease.

Hence, we conducted this meta-analysis with the aim to estimate the prevalence of hypothyroidism among pregnant women in India.

## 2. Methods

This meta-analysis article is written in accordance with the PRISMA guidelines [23] and is registered in the PROSPERO database (CRD42019137955).

### 2.1. Eligibility Criteria

We included the studies reporting the prevalence of hypothyroidism in pregnant women in India.

  Inclusion criteria: (1) community or hospital-based studies; (2) studies, providing prevalence of hypothyroidism (subclinical or overt or category nonspecified); (3) studies conducted in India; (4) type of studies: cross-sectional studies, cohort studies, or data-based analysis; (5) diagnosis based on TSH level; (6) singleton pregnancy
  Exclusion criteria: (1) studies conducted in the special population groups like the diabetic mothers or mothers with pregnancy losses, etc.; (2) studies which have not reported screening methods; (3) diagnosis other than TSH levels; (4) case-control studies or experimental studies

### 2.2. Information Sources

We performed searches in PubMed, Web of Science, and Scopus using selected keywords. These results were supplemented by relevant studies obtained form Google Scholar and Shodhganga—Indian thesis repository (https://shodhganga.inflibnet.ac.in/). We included articles published up to December 2019. No date or language restrictions were imposed. The cross-references of the included studies were explored for additional studies. Keywords were identified with discussion among reviewers, and search query was developed separately for each database.

The main themes of search terms were thyroid (hypothyroidism OR thyroid disorder); geographic area (India); pregnancy (pregnancy OR pregnant OR antenatal OR prenatal); and prevalence (prevalence OR cross-sectional OR incidence OR cohort OR prospective OR longitudinal). To develop a robust search strategy, we used controlled descriptors (such as MeSH terms) and Boolean operators.

### 2.3. Study Selection

Two reviewers (ADG and DD) independently conducted searches on all information sources. Then, all search results were uploaded to Rayyan QCRI online software (https://rayyan.qcri.org). Rayyan QCRI helped in ensuring a systematic and comprehensive search and selection process. A third reviewer (VY) managed Rayyan QCRI software, who identified and removed the duplicate citations and ensured independent review of titles and abstracts by blinding the decisions of both reviewers.

The third reviewer also identified the discrepancies between the two reviewers and discussed them, for making consensus to select the articles. Full-text copies of all selected studies were obtained to find more details. Both reviewers (ADG and DD) reviewed the full text of articles and resolved the discrepancies by consensus. If needed, the arbitration was done by the third reviewer (VY).

**Figure 1:**
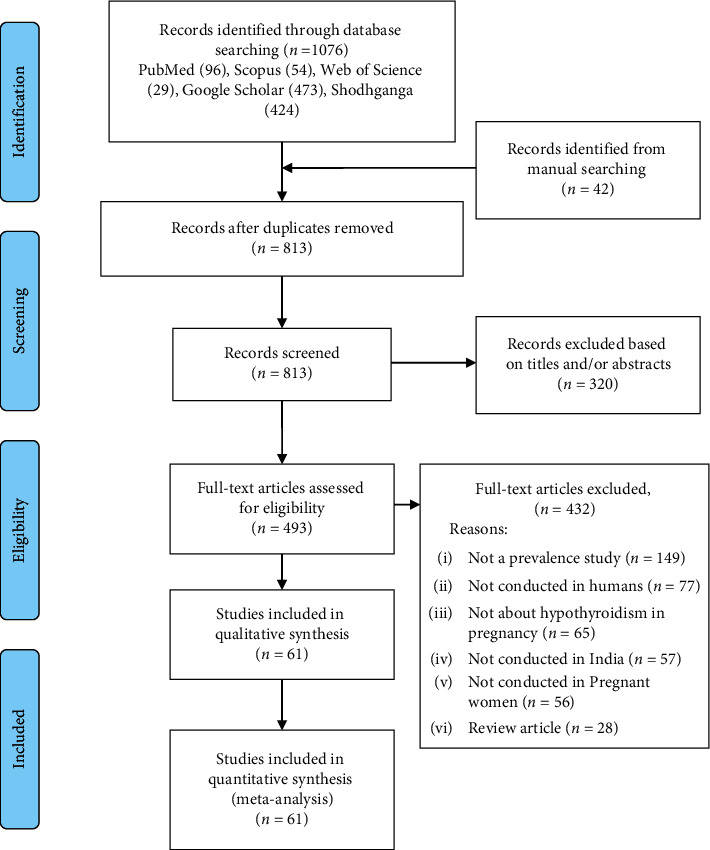
Prisma flowchart.

We documented the reasons for the exclusion of studies explored as full text. If any study is reported as multiple publications, all publications were obtained, and data were extracted from all the publications to collect maximum relevant information. The study inclusion process is presented using the PRISMA flowchart (Figure 1). The reference management software Mendeley Desktop (https://www.mendeley.com) for Windows was used to store, organize, cite, and manage all the included articles.

### 2.4. Data Extraction

After selecting eligible studies, both reviewers (ADG and DD) independently performed data extraction of relevant information. Data were extracted regarding author, year of publication, study location, site (hospital- or community-based or data-based), study type, trimester, whether prepregnancy thyroid disorder patients were excluded, sample size, diagnostic criteria, and prevalence of hypothyroidism (overall, subclinical, and overt). We contacted authors of included articles for additional data whenever required. Inconsistencies in data were resolved by either consensus or seeking further information from the authors of the study. In case of disagreement between two reviewers, arbitration was done by the third reviewer (VY).

### 2.5. Quality Assessment of Studies

Two reviewers (ADG and DD) assessed each study's methodological quality independently using JBI critical appraisal checklist [24] for cross-sectional studies. The components included in this checklist are addressing the target population, appropriateness of participant recruitment, adequacy of sample size, detailed description of study subjects, data analysis with sufficient coverage of the identified sample, use of valid methods for identification of the condition, measurement of the condition in a standardized and reliable way for all participants, use of appropriate statistical analysis, and adequacy of response rate. For any discrepancies, the third reviewer (VY) was consulted. Studies having six or more scores (out of a total of 9) were considered optimum quality studies.

### 2.6. Data Synthesis and Analysis

The effect sizes were calculated for each of the included studies comprising the prevalence estimates of overall hypothyroidism, OH, and SCH among pregnant women in India. Pooled estimates were calculated separately for each of them. All the analysis was done in R software using Meta and Metafor packages [25, 26].

Logit transformation (using Generalized Linear Mixed Models, GLMMs) of proportions was implemented to calculate all pooled estimates, as it is the preferred method for calculating effect size for proportions [27].

Heterogeneity between studies was examined by *I* squared (*I*^2^) statistic and Cochran's *Q* test. Due to significant heterogeneity between the studies, we used random effects models for pooling the estimates and they were reported as a proportion with 95% confidence interval. Restricted Maximum-Likelihood Estimator (REML) method was used to calculate Tau squared. Confidence intervals for individual studies were calculated using the Clopper–Pearson method. We determined the presence of publication bias by visual inspection of funnel plots and rank correlation test. A funnel plot was made between transformed proportions and standard error of transformed proportions. Statistical significance was considered as the *p* value of less than 0.05. Subgroup analysis of estimate of the prevalence of total hypothyroidism was done for “trimester” of participants gestational period (first/second/third/all trimester), whether participants having prepregnancy thyroid disorder were excluded (excluded/not excluded/not mentioned), and the setting of study (hospital-/community-/data-based). India is a federal union that comprises 28 states and 8 union territories. Therefore, state-wise subgroup analysis was also conducted to get state-wise estimates of the prevalence of hypothyroidism. Another subgroup analysis was conducted to analyze the difference of prevalence in studies undertaken in coastal states (Odisha, West Bengal, Gujarat, Kerala, Tamil Nadu, Andhra Pradesh, Maharashtra, Karnataka) as compared to noncoastal states (Assam, Chandigarh, Chhattisgarh, Delhi, Haryana, Jammu and Kashmir, Madhya Pradesh, Manipur, Meghalaya, Punjab, Sikkim, Telangana, Uttar Pradesh, Uttarakhand). Sensitivity analysis was done to exclude studies with a lower sample size (sample size less than 200) and to exclude studies with a quality score of less than six.

## 3. Results

On searching PubMed (*n* = 96), Scopus (*n* = 54), Web of Science (*n* = 29), Google Scholar (*n* = 473), and Shodhganga—reservoir of Indian theses (*n* = 424), a total of 1076 articles were identified related to maternal hypothyroidism.

Google Scholar uses artificial intelligence algorithms to include and rank the articles. It provides only 1000 results for each search query we make. We screened titles of all the 1000 results and found 473 relevant to our purpose.

An additional 42 articles were found eligible for inclusion through search of cross-references. After removal of 305 duplicate articles, a total of 813 articles were screened for inclusion. Based on their titles and abstract, 320 articles were excluded. Thus, the full texts of 493 articles were assessed for eligibility for the systematic review and meta-analysis. The various reasons for exclusion of studies were “not a prevalence study” (*n* = 149), “not conducted in humans” (*n* = 77), “not about hypothyroidism in pregnancy” (*n* = 65), “not conducted in India” (*n* = 57), “not conducted in pregnant women” (*n* = 56), and “review article” (*n* = 28).

All included studies (*n* = 61) provided the prevalence of overall hypothyroidism (total population 60,066) (Figure 1) [6, 7, 28–86]. Among them, 49 studies provided the prevalence of SCH (total population 33,068), and 42 studies provided the prevalence estimates of OH (total population 30,980). Fifty-four studies were conducted in a hospital-based setting, five were secondary data analysis, and two were community-based studies. Almost half (31 studies) of the studies were conducted in coastal states, 29 studies conducted in noncoastal states, and only 1 study conducted in multiples sites involving both coastal and noncoastal states (Table 1).

The definition of overall hypothyroidism (elevated TSH), subclinical hypothyroidism (elevated TSH with normal free thyroid hormone), and overt hypothyroidism (elevated TSH with low free thyroid hormone) was similar across the selected studies, but the reference cutoffs for TSH and free thyroid hormones (FT_4_ and FT_3_) were varied across studies. Upper cutoff limit of TSH for defining hypothyroidism in pregnancy varied from 2.5 to 4.5 mIU/L. For OH, the lower cutoff limits for FT4 and FT3 varied extensively among selected studies (Table 1). Thirty-five studies used American Thyroid Association 2011 (ATA 2011) guidelines for defining hypothyroidism [1] and the rest (26) of the studies used nonstandardized criteria for defining hypothyroidism.

### 3.1. Prevalence Estimates of Hypothyroidism in Pregnant Women in India

**Figure 2:**
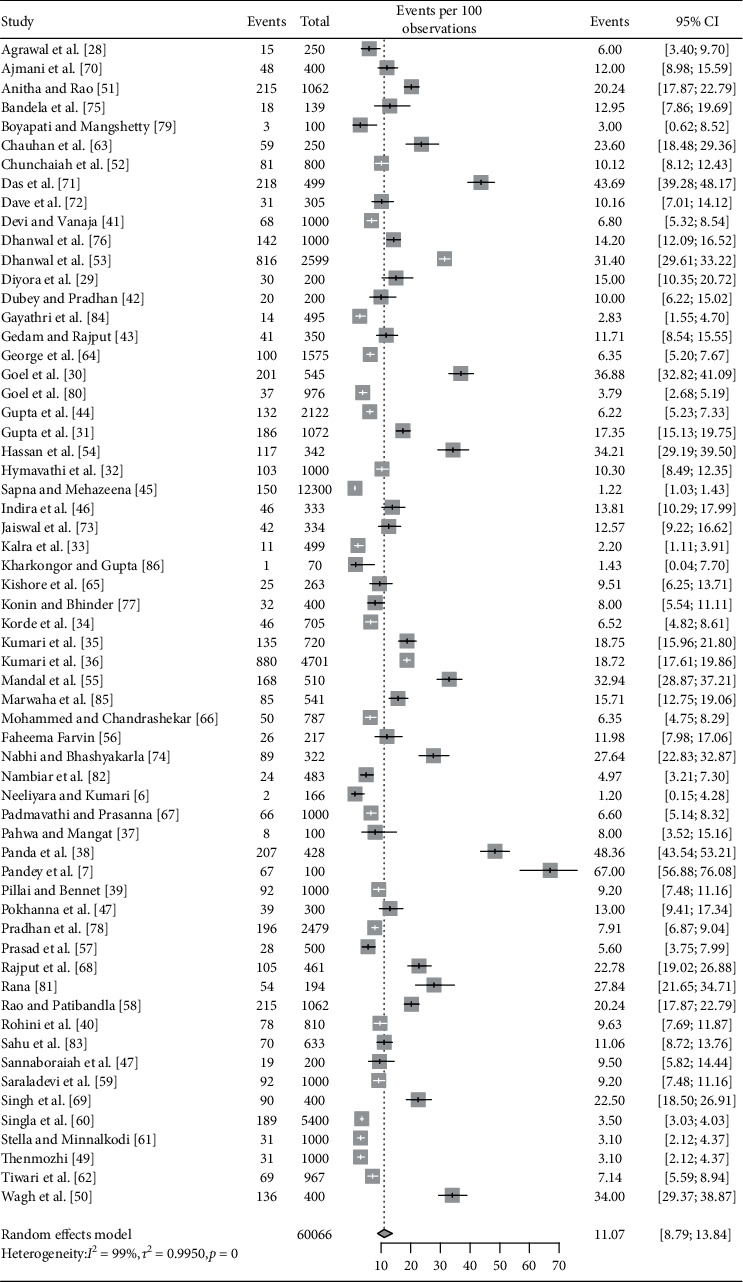
Forest plot showing pooled estimate for prevalence of overall hypothyroidism.

**Figure 3:**
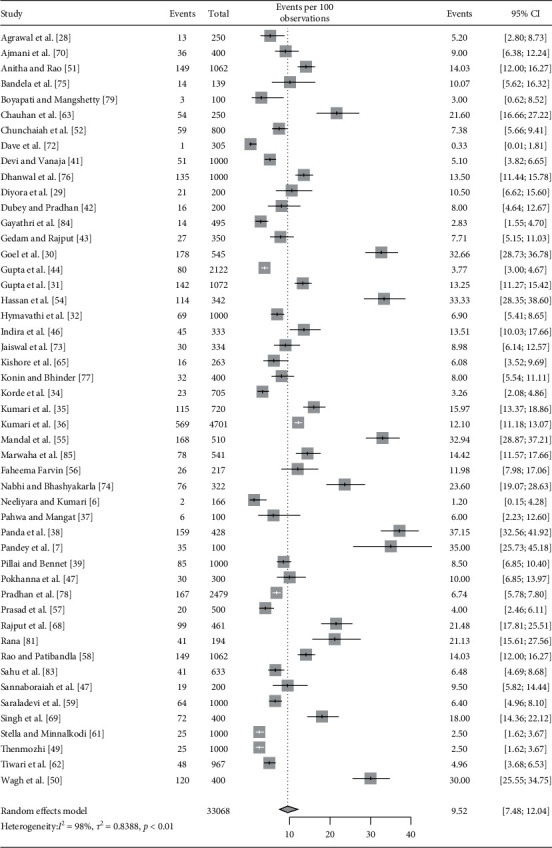
Forest plot showing pooled estimate for prevalence of subclinical hypothyroidism.

**Figure 4:**
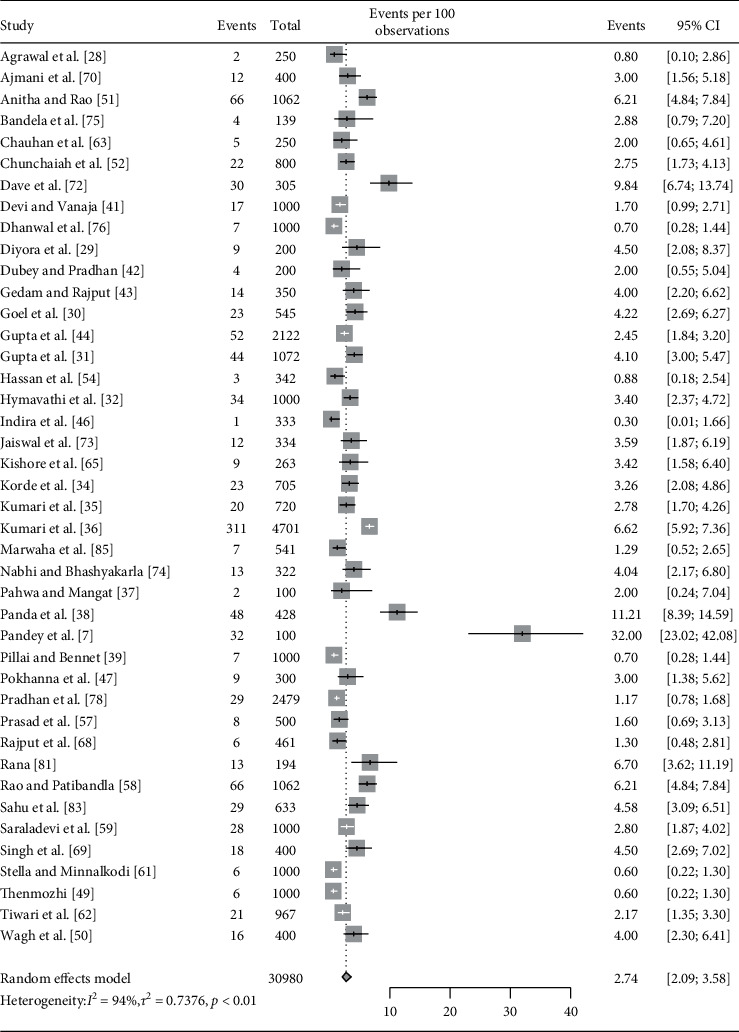
Forest plot showing pooled estimate for prevalence of overt hypothyroidism.

Pooled estimate (with random effects model) of the prevalence of overall hypothyroidism in pregnant women was 11.07% (95% CI: 8.79–13.84, *I*^2^ = 99%). Pooled prevalence estimates of subclinical and overt hypothyroidism were 9.51% (95% CI: 7.48–12.04, *I*^2^ = 98%) and 2.74% (95% CI: 2.08–3.58, *I*^2^ = 94%), respectively (Figures 2–4).

### 3.2. Subgroup Analysis of Overall Hypothyroidism Estimates

Studies that included only women with second-trimester pregnancy documented a higher prevalence of hypothyroidism (25.51, 95% CI: 10.41–50.24) as compared to studies with women in first trimester (10.99, 95% CI: 8.11–14.74), or any trimester of pregnancy (10.05, 95% CI: 7.33–13.65). However, these differences across various subgroups were not statistically significant (*p* = 0.154).

The estimated pooled prevalence was not statistically different among studies that excluded prepregnancy thyroid disorder patients (11.31, 95% CI: 8.57–14.79) as compared to those studies where they were included (10.89, 95% CI: 7.73–15.14) in the analysis.

Studies conducted in hospital settings reported higher pooled prevalence of hypothyroidism among pregnant women (12.32, 95% CI: 9.97–15.13) as compared to secondary data (6.24, 95% CI: 1.83–19.17) and community-based settings (1.27, 95% CI: 0.41–3.86) (*p* < 0.001).

Studies conducted in coastal states documented lower prevalence in comparison to noncoastal states though this difference was not statistically significant (coastal: 8.82, 95% CI: 6.45–11.94 vs. non-coastal:13.49, 95% CI: 9.77–18.34, *p*=0.059) (Table 2).

Studies conducted using ATA 2011 criteria reported higher prevalence (13.71, 95% CI: 10.52–17.67) as compared to studies used other criteria (8.10, 95% CI: 5.49–11.80) for diagnosis and this difference was statistically significant (*p*=0.024).

**Figure 5:**
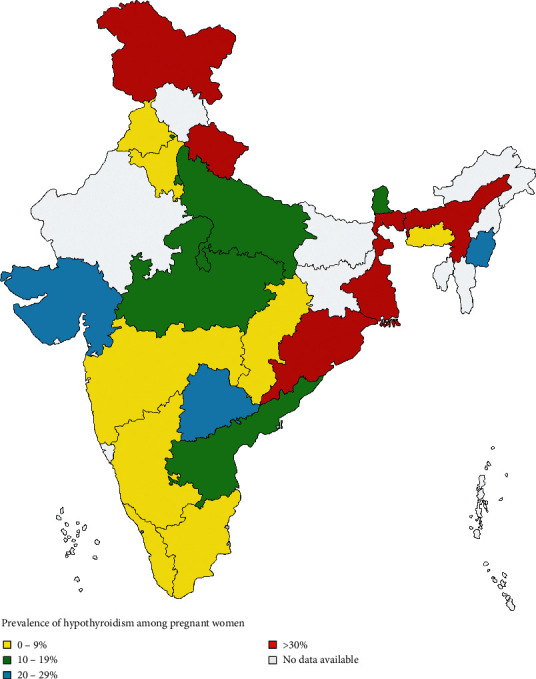
Distribution of hypothyroidism across various states of India.

State-wise pooled prevalence was also calculated and is presented in Table 3 and Figure5.

### 3.3. Sensitivity Analysis of Overall Hypothyroidism Estimates

The sensitivity analysis was conducted by removing lower-quality studies. Prevalence estimate was 10.23 (95% CI 7.41–13.97, *I*^2^:98.4%, 29 studies) after omitting studies with a quality score of less than six on JBI criteria. Another sensitivity analysis was executed to assess the effect of studies with lower sample-sizes on pooled prevalence. This analysis was performed by excluding smaller sample size studies (sample size <200) and pooled prevalence estimate was 11.22 (95% CI: 9.00–13.91, *I*^2^: 98.7%, 54 studies). Both small sample size and low-quality studies did not affect much the prevalence estimate.

### 3.4. Publication Bias

**Figure 6:**
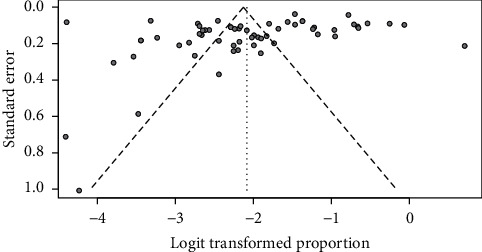
Funnel plot depicting publication bias of included studies.

Regarding the publication bias of pooled estimate of overall hypothyroidism, funnel plot is reasonably symmetrical on visual inspection (Figure 6). Rank correlation test results (*z* = −1.73, *p* value = 0.082) also confirm absence of publication bias.

## 4. Discussion

The sixty-one studies included in the current review have reported prevalence of maternal hypothyroidism ranging from 1.2% to 67.0% [6, 7].

This meta-analysis estimates the prevalence of hypothyroidism in pregnant women in India to be 11.07% (95% CI: 8.79–13.84) from sixty-one studies across 60,066 study subjects. Pooled prevalence estimates of subclinical and overt hypothyroidism were 9.52% (95% CI: 7.48–12.04) and 2.74% (95% CI: 2.09–3.58), respectively.

Sepasi et al. conducted a meta-analysis to report prevalence of hypothyroidism clinical and subclinical hypothyroidism in Iranian pregnant women. Prevalence estimate for hypothyroidism, clinical hypothyroidism, and subclinical hypothyroidism was 13.01% (95% CI: 9.15–18.17), 1.35% (95% CI: 0.97–1.86), and 11.90% (95% CI: 7.40–18.57), respectively, which are very similar to our study [87]. However, prevalence reported in our study is remarkably higher as compared to the reported prevalence of antenatal hypothyroidism in other countries [3–5]. The higher burden in the Indian context may be attributed to the iodine deficiency, prevalent in many regions of the country [88–90].

In this review, we found prevalence of hypothyroidism was lower among coastal areas as compared to studies conducted in noncoastal areas, though difference was nonsignificant (*p*=0.059). Unnikrishnan et al. conducted a survey of hypothyroidism among adult population in 8 urban cities of India [91]. They reported significant lower prevalence of hypothyroidism in coastal cities as compared to noncoastal cities. This may be due to iodine deficiency, which is still more prevalent in noncoastal areas of India [88–90].

In our study, we documented higher prevalence of hypothyroidism in studies that used ATA 2011 criteria as compared to studies that used other criteria, and this difference is statistically significant (*p*=0.024). This may be due to higher cutoffs of TSH for diagnosis, used by other stidies.

Primary maternal hypothyroidism is characterized by an increase in the serum TSH levels during pregnancy. It is further classified as subclinical hypothyroidism (SCH) which has normal free T4 levels and overt hypothyroidism (OH) which has decreased free T4 levels. This differentiation is crucial as it has clinical and management implications [2]. Maternal complications reported to be associated with overt hypothyroidism include pre-eclampsia, placental abruption, polyhydramnios, oligohydramnios, hyperemesis, gestational diabetes, premature rupture of membranes, and chronic hypertension [10–13, 92, 93]. For the fetus too, there is a high risk of fetal death, prematurity, low birth weight, congenital malformations, fetal distress, perinatal hypoxic encephalopathy, and deficit in the mental developmental coefficient [11, 14–19, 93]. Some epidemiological studies have also pointed towards the association of maternal hypothyroidism and adverse neurological outcomes in the progeny ranging from neurological cretinism, congenital hypothyroidism, to decreased intelligence quotient [20–22]. Jansen et al. observed 1981 mother-child pairs [94]. They found that both abnormal (low or high) maternal TSH values, early in pregnancy, were associated with a smaller offspring's total grey matter and cortical volume as assessed by MRI [94].

Data pertaining to effects of early management of SCH in pregnancy are emerging in latest research. Rao et al. in 2019 identified that levothyroxine supplementation significantly decreased pregnancy loss and prematurity in women with SCH [95]. However, Casey et al. conducted a RCT on cases of SCH in pregnancy to determine benefit of SCH treatment in pregnancy on cognitive health of offspring. They did not find significantly better cognitive outcomes in children up to 5 years of age [96]. Thus, there is inconclusive evidence of benefit of treating SCH in pregnancy.

Pregnancy is a state of increased thyroid hormone requirement. The majority (50–85%) of previously hypothyroid women (on treatment) need to increase their dose of thyroid supplements post conception [5]. Pregnancy serves as a stress test for the thyroid gland, which leads to hypothyroidism in iodine deficient women or in those having limited thyroid reserve. Furthermore, risk factors such as geographical disparity (in terms of iodine-deficient regions especially across India), obesity, prior history of thyroid dysfunction, the genetic history of thyroid dysfunction, and history of autoimmune disorders also make pregnant women more susceptible to hypothyroidism [5].

TSH levels during pregnancy are lower as compared to the nonpregnant state. As per American Thyroid Association recommendations 2011 as well as endocrinology society guidelines for pregnant women, TSH levels should be within the limits of 0.1 to 2.5 mIU/L during the first, 0.2–3.0 mIU/L in second, and 0.3 to 3.0 mIU/L in the third trimester [1]. However, these guidelines were revised in 2017, and it was recommended that the first-trimester upper normal limit cutoff should be obtained by deducting “0.5 mIU/L” from prepregnancy TSH value [2]. If this value is unknown, then 4.0 mIU/L should be taken as the upper limit of normal cutoffs.

Optimal TSH cutoffs have been a topic of controversy since long and may have a bearing on prevalence estimates [97]. European Thyroid Association in 2014 recommended that, due to geographic variation in normal TSH and thyroid hormone levels, reference range should be defined for each antenatal hospital after assessment of local population data [98]. ATA 2017 guidelines also supported these recommendations [2]. These guidelines might be the reason for nonstandardized cutoff values are being used by many studies.

However, in Indian setting, the National Guidelines for Screening of Hypothyroidism during Pregnancy 2014 have considered ATA 2011 guidelines for defining cutoffs [99]. A recent article suggested that 4.0 mIU/L as a cutoff as per the revised ATA recommendations may be too high for Indian settings, and intermediate cut-off of 3.00 should be considered [97].

### 4.1. Limitations of the Current Study

A rigorous and comprehensive search strategy was undertaken, which minimizes the possibility of changes in inferences drawn from our study, but there are limitations too. The review exhibits heterogeneity in terms of data. The prevalence varies with the geographic locations, ethnic disparity, and the nutritional intake of the study population. Another aspect that limits our research is the use of nonstandardized TSH cutoffs for diagnosis by studies, as only 35 studies used ATA 2011 TSH cutoffs [1]. Furthermore, preanalytical factors such as gestational age, presence of thyroid antibodies, iodine status, multiple pregnancies, ethnicity, and time of collection of TSH sample may also affect the results. Different immunoassays result in different TSH values, thereby questioning the reliability and repeatability of tests in various studies. Another crucial aspect is the circadian TSH rhythm varying in the second and third trimester of pregnancy. Considering this fact, the standardized collection of samples at the correct time may alter the results and their interpretation.

## 5. Conclusion

This review estimated that every 1 in 10 pregnant Indian women suffers from hypothyroidism. The present study has paved the way for the acceptance of universal screening in pregnant women, especially in the Indian context. The prevalence of hypothyroidism in pregnant women varies across states in India, but data is insufficient. Moreover, there are no agreed-upon guidelines for treating subclinical hypothyroidism in pregnant women. Therefore, further research is needed to fill these gaps regarding the diagnosis and management of hypothyroidism in pregnant women in a heterogeneous country like India.

## Figures and Tables

**Table 1:** Summary of included studies.

S. no	Study (author, year of publication, and study location)	Setting	Definition used (Δ1—first trimester, Δ2—second trimester, Δ3—third trimester)	Hypothyroidism before pregnancy	Participant characteristics (Δ1—first trimester, Δ2—second trimester, Δ3—third trimester)	Total participants number and outcomes
1	Agrawal et al. 2019; New Delhi [28]	Hospital-based	SCH: TSH > 4.5 mIU/L in Δ1, Δ2, Δ3 with normal T4 (>8.5 pmol/L), OH: TSH > 4.5 mIU/L in Δ1, Δ2, Δ3 with low T4 (<8.5 pmol/L)	Included	*n* = 250, age: not mentioned, GA: Δ1	TH: 15 OH: 2 SCH: 13
2	Diyora et al. 2019; Vadodara, Gujarat [29]	Hospital-based	ATA 2011	Excluded	*n* = 200, age: 25.56 ± 3.32 years, GA: Δ1	TH: 30 OH: 9 SCH: 21
3	Goel et al. 2018; Chandigarh [30]	Hospital-based	SCH: TSH > 2.5–10 mIU/L in Δ1, Δ2, Δ3, OH: TSH > 10 mIU/L	Included	*n* = 545, age: 26.21 ± 3.78 years, GA: Δ1 Δ2, Δ3	TH: 201 OH: 23 SCH:178
4	Gupta et al. 2018; Kanpur, Uttar Pradesh [31]	Hospital-based	ATA 2011	Included	*n* = 1072, age: not mentioned, GA: Δ1 Δ2, Δ3	TH: 186 OH: 44 SCH:142
5	Hymavathi et al. 2018; Nellore, Andhra Pradesh [32]	Hospital-based	SCH: TSH > 2.5 mIU/L in Δ1, TSH > 3 mIU/L in Δ2 and TSH > 3.5 mIU/L in Δ3 with normal T4 (cutoffs not mentioned); OH: TSH > 2.5 mIU/L in Δ1, TSH > 3 mIU/L in Δ2 and TSH > 3.5 mIU/L in Δ3 with low T4 (cutoffs not mentioned)	Included	*n* = 1000, age: 24 years, GA: Δ1 Δ2, Δ3	TH: 103 OH: 34 SCH: 69
6	Kalra et al. 2018; Karnal, Haryana [33]	Secondary data from hospital-based study?	Hypothyroidism: TSH > 3 mIU/L in Δ1, Δ2, Δ3	Included	*n* = 499, age: not mentioned, GA: Δ1, Δ2, Δ3	TH: 11
7	Korde et al. 2018; Dabhade, Maharashtra [34]	Hospital-based	ATA 2011	Included	*n* = 705, age: not mentioned, GA: Δ1, Δ2, Δ3	TH: 46 OH: 23 SCH: 23
8	Kumari et al. 2018; Gorakhpur, Uttar Pradesh [35]	Hospital-based	ATA 2011	Excluded	*n* = 720; age: not mentioned; GA: <20 week, Δ1	TH: 135 OH: 20 SCH: 115
9	Kumari et al. 2018; Visakhapatnam, Andhra Pradesh [36]	Hospital-based	ATA 2011	Excluded	*n* = 4701; age: not mentioned; GA: in Δ1, Δ2, Δ3	TH: 880 OH: 311 SCH:569
10	Pahwa and Mangat 2018; Amritsar, Punjab [37]	Hospital-based	ATA 2011	Included	*n* = 100; age: 23.27 ± 4.3 years; GA: Δ1	TH: 8 OH: 2 SCH: 6
11	Panda et al. 2018; Bhubaneswar, Odisha [38]	Hospital-based	SCH: TSH > 2.5–10 mIU/L in Δ1, Δ2, Δ3 with normal T4 (cutoffs not mentioned); OH: TSH > 2.5 mIU/L in Δ1, Δ2, Δ3 with low T4 (cutoffs not mentioned) or TSH > 10 mIU/L	Excluded	*n* = 428; age: 23.95 ± 3.8 years; GA: median 12 weeks (range 6–38), Δ1, Δ2, Δ3	TH: 207 OH: 48 SCH: 159
12	Pandey et al. 2018; Dehradun, Uttarakhand [7]	Hospital-based	ATA 2011	Included	*n* = 100; age: 26.6 ± 4.13 years; GA: Δ2	TH: 67 OH: 32 SCH: 35
13	Pillai and Bennet 2018; Karakonam, Kerala [39]	Hospital-based	ATA 2011	Excluded	*n* = 1000; age: not mentioned; GA: Δ1, Δ2, Δ3	TH: 92 OH: 7 SCH: 85
14	Rohini et al. 2018; Bengaluru, Karnataka [40]	Hospital-based	ATA 2011	Excluded	*n* = 810; age: 27.2 years; GA: Δ1, Δ2, Δ3	TH: 78 OH: SCH:
15	Devi and Vanaja 2017; Visakhapatnam, Andhra Pradesh [41]	Hospital-based	SCH: TSH 3–10 mIU/L in Δ1, Δ2, Δ3 with normal T4 (cutoffs not mentioned); OH: TSH > 3 mIU/L in Δ1, Δ2, Δ3 with low T4 (cutoffs not mentioned) or TSH > 10 mIU/L	Excluded	*n* = 1000; age: not mentioned; GA: <12 week, Δ1	TH: 68 OH: 17 SCH: 51
16	Dubey and Pradhan 2017; Gangtok, Sikkim [42]	Hospital-based	ATA 2011	Excluded	*n* = 200; age: 20–35 years; GA: 6–24 weeks, Δ1, Δ2, Δ3	TH: 20 OH: 4 SCH: 16
17	Gedam and Rajput 2017; Mumbai, Maharashtra [43]	Hospital-based	ATA 2011	Excluded	*n* = 350; age: not mentioned; GA: Δ1, Δ2, Δ3	TH: 41 OH: 14 SCH: 27
18	Gupta et al. 2017; Patiala, Punjab [44]	Hospital-based	SCH: TSH > 3–6 mIU/L in Δ1, Δ2, Δ3; OH (clinical hypothyroidism): TSH > 6 mIU/L in Δ1, Δ2, Δ3	Included	*n* = 2122; age: not mentioned; GA: Δ1, Δ2, Δ3	TH: 132 OH: 52 SCH: 80
19	Sapna and Mehazeena 2017; Mumbai, Maharashtra [45]	Secondary data	Hypothyroidism: TSH > 3 mIU/L in Δ1, Δ2, Δ3	Included	*n* = 12300; age: not mentioned; GA: Δ1, Δ2, Δ3	TH: 150 OH: SCH:
20	Indira et al. 2017; Vizianagaram, Andhra Pradesh [46]	Hospital-based	ATA 2011	Included	*n* = 333; age: not mentioned; GA: Δ1, Δ2, Δ3	TH: 46 OH: 1 SCH: 45
21	Pokhanna et al. 2017; Indore, Madhya Pradesh [47]	Hospital-based	ATA 2011	Excluded	*n* = 300; age: not mentioned; GA: 13–26 weeks, Δ2	TH: 39 OH: 9 SCH: 30
22	Sannaboraiah et al. 2017; not provided, Karnataka [48]	Hospital-based	SCH: TSH > 2.5 mIU/L in Δ1 and > 3 mIU/L in Δ2 and > 3.5 mIU/L in Δ3 with normal T3, T4 (cutoffs not mentioned)	Excluded	*n* = 200; age: not mentioned; GA: Δ1, Δ2, Δ3	TH: 19 SCH: 19
23	Thenmozhi 2017; Kancheepuram, Tamil Nadu [49]	Hospital-based	ATA 2011	Included	*n* = 1000; age: not mentioned; GA: Δ1, Δ2, Δ3	TH: 31 OH: 6 SCH: 25
24	Wagh et al. 2017; Nagpur, Maharashtra [50]	Secondary data	ATA 2011	Included	*n* = 400; age: not mentioned; GA: Δ1, Δ2, Δ3	TH: 136 OH: 16 SCH: 120
25	Anitha and Rao 2016; Hyderabad, Telangana [51]	Hospital-based	ATA 2011	Included	*n* = 1062; age: not mentioned; GA: Δ1, Δ2, Δ3	TH: 215 OH: 66 SCH: 149
26	Chunchaiah et al. 2016; Banguluru, Karnataka [52]	Hospital-based	ATA 2011	Included	*n* = 800; age: not mentioned; GA: Δ1, Δ2, Δ3	TH: 81 OH: 22 SCH: 59
27	Dhanwal et al. 2016; Allahabad, Bengaluru, Chennai, Kolkata, Hyderabad, Nasik, Rohtak, Pune, New Delhi, Srinagar, and Vizag,Uttar Pradesh, Karnataka, Tamil Nadu, West Bengal, Maharashtra, Haryana, Maharashtra, Delhi, Kashmir and Andhra Pradesh [53]	Hospital-based	Hypothyroidism: TSH > 4.5 mIU/L in Δ1, Δ2, Δ3	Included	*n* = 2599; age: 25.5 ± 5.6 years; GA: 19.3 ± 15.9 weeks, Δ1, Δ2, Δ3	TH: 816 OH: SCH:
28	Hassan et al. 2016; J & K, Srinagar [54]	Hospital-based	ATA 2011	Included	*n* = 342; age: not mentioned; GA: Δ1, Δ2, Δ3	TH: 117 OH: 3 SCH: 114
29	Mandal et al. 2016; Kolkata, West Bengal [55]	Hospital-based	ATA 2011	Excluded	*n* = 510; age: 18.7 ± 3.52 years; GA: 7.6 ± 1.12 weeks, Δ1	TH: 168 OH: SCH:168
30	Faheema Farvin 2016; Chennai, Tamil Nadu [56]	Hospital-based	SCH: TSH > 3 mIU/L in Δ1, Δ2, Δ3 with normal T4 (11.84 ± 3.86 pmol/L); OH: TSH > 3 mIU/L in Δ1, Δ2, Δ3 with low T4 (<11.84 pmol/L) or TSH > 10 mIU/L	Excluded	*n* = 217; age: 27.5 ± 2.12 years; GA: Δ1, Δ2, Δ3	TH: 26 OH: SCH: 26
31	Prasad et al. 2016; Trivandrum, Kerala [57]	Hospital-based	ATA 2011	Included	*n* = 500; age: 25.4 ± 3.7 years; GA: Δ1	TH: 28 OH: 8 SCH: 20
32	Rao and Patibandla 2016; Hyderabad, Telangana [58]	Secondary data	ATA 2011	Included	*n* = 1062; age: 22.1 years; GA: Δ1, Δ2, Δ3	TH: 215 OH: 66 SCH: 149
33	Saraladevi et al. 2016; Warangal, Maharashtra [59]	Hospital-based	ATA 2011	Included	*n* = 1000; age: not mentioned; GA: Δ1	TH: 92 OH: 28 SCH: 64
34	Singla et al. 2016; Ludhiana, Punjab [60]	Secondary data	ATA 2011	Included	*n* = 5400; age: not mentioned; GA: Δ1, Δ2, Δ3	TH: 189 OH: SCH:
35	Stella and Minnalkodi 2016; Chennai, Tamil Nadu [61]	Hospital-based	ATA 2011	Included	*n* = 1000; age: not mentioned; GA: Δ1	TH: 31 OH: 6 SCH: 25
36	Tiwari et al. 2016; New Delhi, Delhi [62]	Hospital-based	ATA 2011	Excluded	*n* = 967; age: not mentioned; GA: Δ1, Δ2, Δ3	TH: 69 OH: 21 SCH: 48
37	Chauhan et al. 2015; Jabalpur, Madhya Pradesh [63]	Hospital-based	ATA 2011	Included	*n* = 250; age: majority 20–30 years; GA: Δ1, Δ2, Δ3	TH: 59 OH: 5 SCH: 54
38	George et al. 2015; Kochi, Kerala [64]	Hospital-based	ATA 2011	Included	*n* = 1575; age: not mentioned; GA: Δ1, Δ2, Δ3	TH: 100 OH: SCH:
39	Kishore et al. 2015; Raipur, Chhattisgarh [65]	Hospital-based	ATA 2011	Included	*n* = 263; age: not mentioned; GA: Δ1	TH: 25 OH: 9 SCH: 16
40	Mohammed and Chandrashekar 2015; Bellary, Karnataka [66]	Hospital-based	ATA 2011	Included	*n* = 787; age: not mentioned; GA: Δ1, Δ2, Δ3	TH: 50 OH: SCH:
41	Neeliyara and Kumari 2015; Alleppey, Kerala [6]	Community-based	SCH: TSH > 4.5 mIU/L in Δ1, Δ2, Δ3 with normal T4 (>0.62 ng/dl); OH: TSH > 4.5 mIU/L in Δ1, Δ2, Δ3 with low T4 (<0.62 ng/dl)	Included	*n* = 166; age: not mentioned; GA: Δ1, Δ2, Δ3	TH: 2 OH: 0 SCH: 2
42	Padmavathi and Prasanna 2015; Visakhapatnam, Andhra Pradesh [67]	Hospital-based	Hypothyroidism: TSH more than 2.3 mIU/L/L, 3.7 mIU/L, 3.4 mIU/L in Δ1, Δ2, and Δ3, respectively	Excluded	*n* = 1000; age: not mentioned; GA: <12 week, Δ1	TH: 66 OH: SCH:
43	Rajput et al. 2015; Rohtak, Haryana [68]	Hospital-based	ATA 2011	Excluded	*n* = 461; age: 23.79 ± 3.47 years; GA: 8 weeks 5 days., Δ1	TH: 105 OH: 6 SCH: 99
44	Singh et al. 2015; Imphal, Manipur [69]	Hospital-based	SCH: TSH > 3 mIU/L in Δ1, Δ2, Δ3 with normal T4 (>7.5 mcg/dl); OH: TSH > 3 mIU/L in Δ1, Δ2, Δ3 with Low T4 (<7.5 mcg/dl)	Excluded	*n* = 400; age: 26.8 ± 8.2 years; GA: Δ1, Δ2, Δ3	TH: 90 OH: 18 SCH: 72
45	Ajmani et al. 2014; Delhi, Delhi [70]	Hospital-based	SCH: TSH > 3 mIU/L in Δ1, Δ2, Δ3 with normal T4 (0.8–2 ng/dl); OH: TSH > 3 mIU/L with low T4 (<0.8 ng/dl)	Excluded	*n* = 400; age: not mentioned; GA: 13–26 weeks, Δ1	TH: 48 OH: 12 SCH: 36
46	Das et al. 2014; Joti Gaon, Assam [71]	Hospital-based	ATA 2011	Included	*n* = 499; age: 18–35 years; GA: Δ1	TH: 218 OH: SCH:
47	Dave et al. 2014; Indore, Madhya Pradesh [72]	Hospital-based	ATA 2011	Included	*n* = 305; age: 24.46 (2) years; GA: 9.09 weeks, Δ1	TH: 31 OH: 30 SCH: 1
48	Jaiswal et al. 2014; Banglore, Karnataka [73]	Hospital-based	ATA 2011	Excluded	*n* = 334; age: not mentioned; GA: 10.3 ± 2.5 weeks, Δ1	TH: 42 OH: 12 SCH: 30
49	Nabhi and Bhashyakarla 2014; Hyderabad, Telangana [74]	Hospital-based	ATA 2011	Included	*n* = 322; age: not mentioned; GA: Δ2	TH: 89 OH: 13 SCH: 76
50	Bandela et al. 2013; Nandyal, Andhra Pradesh [75]	Hospital-based	SCH: TSH 4–10 mIU/L in Δ1, Δ2, Δ3 with normal T4 (cutoffs not mentioned); OH: TSH 4–10 mIU/L in Δ1, Δ2, Δ3 with low T4 (cutoffs not mentioned) or TSH > 10 mIU/L	Included	*n* = 139; age: median 25 years, 17–35 years; GA: only <13 week included, median 8.5 weeks, Δ1	TH: 18 OH: 4 SCH: 14
51	Dhanwal et al. 2013; New Delhi, Delhi [76]	Hospital-based	SCH: TSH > 4.5 mIU/L in Δ1, Δ2, Δ3 with normal T3, T4 (cutoffs not mentioned); OH: TSH > 4.5 mIU/L with low T3, T4 (cut-offs not mentioned); thyrotoxicosis: TSH > 150 mIU/L	Included	*n* = 1000; age: 25.6 ± 11.1 years; GA: 9.2 ± 3.4 weeks, Δ1	TH: 142 OH: 7 SCH: 135
52	Konin and Bhinder 2013; Gulbarga, Karnataka [77]	Hospital-based	SCH: TSH > 4.2 mIU/L in Δ1, Δ2, Δ3 with normal FT4 (0.80–2.7 ng/dl) and FT3 (2–3.8 pg/mL); OH: TSH > 4.2 mIU/L with low FT4 (<0.80 ng/dl) and FT3 (<2 pg/mL)	Excluded	*n* = 400; age: not mentioned; GA: ≤14 weeks, Δ1	TH: 32 OH: 0 SCH: 32
53	Pradhan et al. 2013; Lucknow, Uttar Pradesh [78]	Hospital-based	SCH: TSH > 2.5 mIU/L in Δ1, Δ2, Δ3 with normal T3, T4 (cutoffs not mentioned); OH: TSH > 2.5 mIU/L with low T3, T4 (cut-offs not mentioned)	Included	*n* = 2479; age: mostly 20–40, 2 < 20 yr, 2 > 40 years; GA: Δ1, Δ2, Δ3	TH: 196 OH: 29 SCH: 167
54	Boyapati and Magshetty 2012; Gulbarga, Karnataka [79]	Hospital-based	SCH: TSH > 5 mIU/L in Δ1, Δ2, Δ3 with normal T4 (0.93–1.7 ng/dl); OH: TSH > 4.5 mIU/L in Δ1, Δ2, Δ3 with low T4 (<0.93 ng/dl)	Excluded	*n* = 100; age: not mentioned; GA: Δ1, Δ2, Δ3	TH: 3 OH: SCH: 3
55	Goel et al. 2012; Chandigarh, Chandigarh [80]	Hospital-based	Hypothyroidism: TSH > 5.5 mIU/L in Δ1, Δ2, Δ3	Excluded	*n* = 976; age: known hyothyroid: 27.6 ± 2.9, new hypothyroid: 27.5 ± 4.1, euthyroid group: 25.5 ± 3.9; GA: known hyothyroid: 19.5 ± 9.4, new hypothyroid: 20.3 ± 8.9, euthyroid group: 19.6 ± 7.8, Δ1, Δ2, Δ3	TH: 37 OH: SCH:
56	Rana 2012; Vadodara, Gujarat [81]	Hospital-based	ATA 2011	Included	*n* = 194; age: 23.3 (3.6) years and; GA: Δ1, Δ2, Δ3	TH: 54 OH: 13 SCH: 41
57	Nambiar et al. 2011; Mumbai, Maharashtra [82]	Hospital-based	Hypothyroidism: TSH > 4 mIU/L in Δ1, Δ2, Δ3	Excluded	*n* = 483; age: 25.19 ± 4.17 years; GA: 10.03 ± 1.87 weeks, Δ1	TH: 24 OH: SCH:
58	Sahu et al. 2010; Lucknow and New Delhi, Uttar Pradesh and New Delhi [83]	Hospital-based	SCH: TSH > 5.5 mIU/L in Δ1, Δ2, Δ3 with normal T3, T4 (cutoffs not mentioned); OH: TSH > 5.5 mIU/L with low T3, T4 (cutoffs not mentioned)	Included	*n* = 633; age: not mentioned; GA: 13–26 weeks, Δ2	TH: 70 OH: 29 SCH: 41
59	Gayathri et al. 2009; Chennai, Tamil Nadu [84]	Hospital-based	SCH: TSH > 5 mIU/L in Δ1, Δ2, Δ3 with normal T3, T4 (cutoffs not mentioned)	Excluded	*n* = 495; age: 23.8 ± 3.7 years; GA: Δ1, Δ2, Δ3	TH: 14 OH: SCH: 14
60	Marwaha et al. 2008; Delhi, Delhi [85]	Hospital-based	SCH: TSH > 4.2 mIU/L in Δ1, Δ2, Δ3 with normal T3 (3.7–7.2 pg/ml), T4 (12.0–23.0 pg/ml); OH: TSH > 4.2 mIU/L in Δ1, Δ2, Δ3 with normal T3 (<3.7 pg/ml), T4 (<12.0 pg/ml)	Excluded	*n* = 541; age: not mentioned; GA: Δ1, Δ2, Δ3	TH: 85 OH: 7 SCH: 78
61	Kharkongor and Gupta 1998; Meghalaya [86]	Community-based	Hypothyroidism: TSH > 7 mIU/L in Δ1, Δ2, Δ3	Included	*n* = 70; age:15–25 years; GA: Δ1, Δ2, Δ3	TH: 1 OH: SCH:

ATA: American Thoracic Association; TH: total hypothyroidism; OH: overt hypothyroidism; SCH: subclinical hypothyroidism.

**Table 2:** Subgroup analysis of overall hypothyroidism estimates.

Subgroup categories	Number of studies	ES	95% CI	*I * ^2^ (%)	Between group *Q*	Between group *p* value
Gestational period (trimester)					3.47	0.1544
First	20	10.99	8.11–14.74	96.6		
Second	4	25.51	10.41–50.24	98.1		
Any trimester	37	10.05	7.33–13.65	99.1		

Prepregnancy thyroid patients' exclusion					0.03	0.8637
Excluded	25	11.31	8.57–14.79	97.6		
Included	36	10.89	7.73–15.14	99.1		

Site					17.17	<0.001
Hospital-based	54	12.32	9.97–15.13	98.4		
Secondary data	5	6.24	1.83–19.17	99.6		
Community-based	2	1.27	0.41–3.86	0.0		

Coastal state					3.55	0.0597
Yes	31	8.82	6.45–11.94	98.3		
No	29	13.49	9.77–18.34	98.7		

Criteria/definition					5.04	0.0247
ATA 2011	35	13.71	10.52–17.67	98.5		
Other	26	8.10	5.49–11.80	98.8		

**Table 3:** Subgroup analysis showing state-wise prevalence (Cochrane *Q*: 747.87, *p* value: <0.001).

States of India	Number of studies	ES (percent prevalence)	95% CI
Andhra Pradesh	6	10.78	7.80–14.72
Assam	1	43.69	39.39–48.08
Chandigarh	2	13.18	2.27–49.79
Chhattisgarh	1	9.51	6.50–13.69
Delhi	5	10.59	7.62–14.53
Gujarat	2	20.82	13.24–31.19
Haryana	2	7.60	1.33–33.38
Karnataka	7	8.70	7.15–10.53
Kerala	4	5.46	3.15–9.29
Madhya Pradesh	3	14.82	9.66–22.06
Maharashtra	6	7.55	3.21–16.74
Manipur	1	22.5	18.67–26.85
Meghalaya	1	1.43	0.20–9.45
Multiple states	2	19.38	8.85–37.32
Odisha	1	48.36	43.66–53.1
Punjab	3	5.07	3.42–7.47
Sikkim	1	10	6.54–14.99
Jammu and Kashmir	1	34.21	29.37–39.4
Tamil Nadu	4	4.23	2.3–7.64
Telangana	3	22.01	18.82–25.56
Uttar Pradesh	3	13.79	8.80–20.97
Uttarakhand	1	67	57.23–75.49
West Bengal	1	32.94	29.0–37.14

## Data Availability

The data used to support the findings of this study are included within the article as tables.
